# Editorial: Natural bioactive compounds in food preservation and safety

**DOI:** 10.3389/fnut.2025.1720905

**Published:** 2025-11-18

**Authors:** Ignacio Cabezudo, Micaela Galante, Maria Emilia Brassesco, Carolina Beres, Ana Elizabeth Cavalcante Fai

**Affiliations:** 1 Pharmacognosy, Faculty of Biochemical and Pharmaceutical Sciences (FBioyF), National University of Rosario (UNR) and National Council for Scientific and Technical Research (CONICET), Rosario, Argentina; 2 Food Research, Development, and Evaluation Laboratory (LIDEA), Faculty of Biochemical and Pharmaceutical Sciences (FBioyF), National University of Rosario (UNR) and National Council for Scientific and Technical Research (CONICET), Rosario, Argentina; 3 Portuguese Catholic University, CBQF - Center for Biotechnology and Fine Chemistry - Associated Laboratory, School of Biotechnology, Porto, Portugal; 4 Food and Nutrition Graduate Program, Federal University of Rio de Janeiro State (UNIRIO), Rio de Janeiro, Brazil; 5 Department of Basic and Experimental Nutrition, Institute of Nutrition, Rio de Janeiro State University (UERJ), Rio de Janeiro, Brazil

**Keywords:** natural bioactive compounds, food preservation, antimicrobial agents, waste valorization, edible coatings

## Introduction

The global food industry faces unprecedented challenges in ensuring food safety while meeting consumer demands for natural, sustainable preservation solutions. With approximately 600 million cases of foodborne illnesses annually and growing concerns about synthetic additives, the search for natural bioactive compounds has become a priority. This Research Topic brings together innovative approaches that harness nature's arsenal of antimicrobial and antioxidant compounds, demonstrating how agricultural by-products, traditional plant extracts, and microbial metabolites can revolutionize food preservation while addressing sustainability goals.

## Circular economy meets food preservation

A remarkable theme emerging from this Research Topic is the valorization of agricultural waste streams. The article “*Assessment of chitosan based edible coatings containing bioactive compounds derived from agricultural residue for improving postharvest quality characteristics of tomato (Solanum lycopersicum L.)”* exemplifies this approach by transforming rice and wheat straw—typically considered agricultural residue—into potent preservative extracts (Yadav et al.). When incorporated into chitosan-based edible coatings, these extracts significantly extended tomato shelf life, with wheat straw extracts reducing disease incidence from 100% in controls to just 2% after 30 days. Similarly, “*Grape pomace as a natural source of antimicrobial agents for food preservation,”* provides a comprehensive analysis of grape pomace utilization, revealing how this winemaking by-product, rich in phenolic compounds, offers multifaceted applications from direct antimicrobial additives to active packaging materials (Galante et al.). These studies demonstrate that waste valorization not only addresses environmental concerns but also provides practical and economically viable preservation solutions.

## Synergistic formulations and novel delivery systems

sThe collection highlights the power of combining bioactive compounds for enhanced efficacy: “*Effect of chitosan edible coating containing anthocyanins and tea polyphenols on cold storage of chilled pork”* evidences this synergy by combining blackberry anthocyanins with tea polyphenols in chitosan coatings for chilled pork preservation (Chen et al.). The combined treatment achieved superior results compared to individual compounds, reducing total viable counts by 9.3% and lipid oxidation markers by 45.5%. Meanwhile, “*Enhanced antibacterial potential of exopolysaccharide-stabilized spice oil emulsions against foodborne pathogens”* introduces an innovative approach using exopolysaccharide-stabilized emulsions of African spice oils, demonstrating how traditional ingredients can be reformulated and modernized through advanced delivery systems (Kumari Singh et al.). The sonication-enhanced emulsions showed remarkable antibacterial activity against major foodborne pathogens including *Listeria monocytogenes* and *Salmonella enterica*.

## Beyond traditional Preservation: Quality enhancement

A particularly novel contribution comes from “γ*-Aminobutyric acid treatment maintains the quality and improve antioxidative activities of fresh-cut Euryale ferox stems during postharvest storage,”* which explores γ-aminobutyric acid treatment for fresh-cut vegetables (Wang et al.). Unlike conventional preservatives that merely inhibit deterioration, it actively enhanced quality parameters while maintaining antioxidant enzyme activities. This multifunctional approach, simultaneously improving sensory attributes, nutritional value, and shelf life, represents a paradigm shift in preservation strategies. The treatment extended the commercial viability of fresh-cut Euryale ferox stems from 16 to 20 days, demonstrating the compound's potential as a quality-enhancing preservative.

## Future perspectives

This Research Topic highlights several promising directions for natural food preservation. The successful integration of waste-derived compounds into commercial preservation systems could transform both the food and agricultural sectors in line with circular economy principles. However, challenges remain in standardizing extraction protocols, understanding structure-activity relationships, and scaling production. The demonstrated synergies between different bioactive compounds suggest that future research should focus on optimized combinations rather than single-compound solutions.

The convergence of sustainability imperatives, consumer preferences, and technological advances positions natural bioactive compounds at the forefront of food preservation innovation. This Research Topic provides compelling evidence that nature-based solutions can meet or exceed the performance of synthetic preservatives while offering additional benefits including waste reduction, enhanced nutritional value, and improved sustainability profiles. As we advance toward more resilient food systems, the integration of these bioactive compounds into mainstream preservation practices appears not just promising but essential.

